# A pilot randomised controlled trial of the feasibility of using body scan and isometric exercises for reducing urge to smoke in a smoking cessation clinic

**DOI:** 10.1186/1471-2458-8-349

**Published:** 2008-10-06

**Authors:** Lemees Al-Chalabi, Neha Prasad, Lucy Steed, Sarah Stenner, Paul Aveyard, Jane Beach, Michael Ussher

**Affiliations:** 1 Primary Care Clinical Sciences, University of Birmingham, Birmingham, UK; 2 Stop Smoking Service, South Birmingham Primary Care Trust, Birmingham, UK; 3 Division of Community Health Sciences, St George's, University of London, London, UK; 4 NIHR Career Scientist, Primary Care Clinical Sciences, University of Birmingham, Birmingham, B15 2TT, UK

## Abstract

**Background:**

The main cause of relapse in smokers attempting to quit is inability to resist urges to smoke. Pharmacotherapy ameliorates but does not entirely prevent urges to smoke when abstinent, so other methods to resist urges to smoke might be helpful. Exercise is effective, but aerobic exercise is often impractical when urges strike. Two techniques, body scan and isometric exercise, have been shown to reduce urge intensity and nicotine withdrawal symptoms in temporarily abstinent smokers. It is unclear whether they would be used or effective in typical smokers attempting to quit.

**Methods:**

In a pilot trial set in a UK smoking cessation clinic, 20 smokers were randomised to receive emails containing .mp3 files and .pdf illustrations of the instructions for doing the body scan and isometric exercises. Twenty smokers received no other intervention, although all 40 were receiving weekly behavioural support and nicotine replacement therapy. Carbon monoxide confirmed abstinence, nicotine withdrawal symptoms, urges to smoke, and use of the techniques to resist urges were recorded weekly for four weeks after quit day.

**Results:**

60–80% of quitters reported using the isometric exercises each week and 40–70% reported using the body scan to deal with urges. On average, these techniques were rated as 'slightly helpful' for controlling the urges. There were no large or significant differences in withdrawal symptoms or urge intensity between the two groups. The risk ratio and 95% confidence interval for exercises compared with controls for prolonged confirmed abstinence at four weeks was 0.82 (0.44–1.53). 81% of quitters intended to continue using isometric exercises and 25% body scan, while 81% and 50% respectively would recommend using these techniques to others trying to stop.

**Conclusion:**

Isometric exercises, and to a lesser extent body scan, were popular and perceived as somewhat helpful by quitters. The trial showed that these techniques were used and a larger trial could now be developed to examine the influence of the methods on reducing urges to smoke and increasing abstinence.

**Trial registration:**

ISRCTN70036823

## Background

Optimal smoking cessation treatment involves a combination of behavioural support and pharmacotherapy[1]. The behavioural support method used in the UK is called withdrawal orientated therapy and it aims to bolster a person's resolve to endure the immediate period after stopping smoking when urges to smoke and unpleasant psychological and physical symptoms are at their peak[2]. Although behavioural support and pharmacotherapy appear roughly equally effective and their effects are probably multiplicative [3-7], pharmacotherapy research is more common than research in new methods of behavioural intervention to support smoking cessation.

Inability to resist urges to smoke account for many quitters relapsing[8]. It is common in behavioural support to hear therapists advocate the four-Ds as a means to cope with urges to smoke. The four-Ds are delay, deep breathing, drink water, and distract, but none of these methods have evidence to support or refute the assertion that they are effective at reducing urges or helping quitters to resist them[9]. One behavioural intervention that does reduce urges to smoke and nicotine withdrawal symptoms is exercise. A systematic review showed that all 12 studies comparing a bout of exercise with a passive condition reported a positive effect on urges to smoke and withdrawal symptoms[10]. While aerobic activity is useful and can be promoted for a variety of reasons while stopping smoking, it is not always possible for smokers to do aerobic activity when urges strike. Isometric exercise, which involves static muscle contractions (e.g. fist clenching), can be done at a workstation and has also been shown to be effective in reducing urges to smoke and withdrawal in smokers who abstained from smoking for about 15 hours[11]. Another technique, body scan, is a relaxation technique based on mindfulness of one's body and one's breathing. This technique is also practical in many situations and has been shown to reduce urges to smoke and withdrawal in smokers who abstained for around 15 hours[12]. In both these studies, the effects of isometric exercise and body scan in reducing urges to smoke were similar in size to those observed when nicotine replacement therapy (NRT) was used in similar paradigms.

Following the example of NRT, large trials would be needed to show whether these techniques have clinically useful effects enhancing smoking cessation. Before such trials, it would be essential to show supportive data that the techniques are popular and perceived as useful by typical smokers trying to quit, following advice about developing complex interventions[13]. Thus far, such methods have only ever been tested in temporarily abstinent smokers being paid to test the methods. We therefore tested the accessibility of offering these methods by email, acceptability, and perceived usefulness of these methods in a typical smoking cessation clinic. We adopted a pragmatic design, meaning that we allowed natural variation in clinical practice of smoking cessation treatment and did not use a sham relaxation exercise control[14]. We report here effect sizes and their uncertainty and measures of distribution of quantitative variables that would be useful in planning a definitive trial. This trial was not large enough to definitively exclude clinically worthwhile effects.

## Methods

The study was approved by South Birmingham NHS Research Ethics Committee and the National Health Service (NHS) Research and Development Department. Smokers were recruited from NHS stop smoking clinics and which were not run specifically for people interested in using these techniques, nor did their normal clinic protocols differ for this research study. Researchers attended some of these clinics and presented details of the study to clinic attendees where time allowed. Anyone attending the clinic was eligible for recruitment. To be included, participants had to have a current email address and be willing to do the exercises if randomised to them. There were no exclusion criteria. The clinics provide weekly support in a 'drop-in' format. This means that they ran weekly at the same time in the same place over several hours, and participants were free to attend initially without formal referral or appointment and re-attend without booking an appointment.

In the clinic, participants were given NRT for a quit day negotiated with the clinic staff, between the first and second sessions, typically the day of initial attendance or the next day. Participants chose NRT preparations from all licensed formulations with the advice of the stop smoking advisor. The main product used was the patch. Some participants changed preparations and a few used a combination of patch plus oral NRT. The NRT was dispensed in weekly aliquots and the cost was covered by the UK NHS. (In the UK, some people aged 16–59 years with sufficient income pay a small flat rate fee unrelated to the cost of medication for each prescription and this applied to participants in this study). Participants were seen until four weeks after quit day and four weekly thereafter with medication continuing for 8–12 weeks in total. Our trial ceased data collection at 4 weeks after quit day.

We used MS Excel to set up block randomisation with blocks of two. Once a participant agreed to participate, we allocated them to either the intervention (isometric exercise and body scan) or control (no intervention) using the next number in sequence. This sequence was unknown to the recruiters or participants at recruitment. We sent participants an email containing information about their allocation after the baseline visit.

Participants in the intervention group received a .pdf file with pictures of how to do the isometric exercises and corresponding instructions, and two .mp3 files with audio instructions for performing the body scan and the isometric exercises. A participant listening to the .mp3 file and performing either exercise would take ten minutes to complete the exercise. The isometric exercises involved six static muscular contractions that were carried out for three seconds while the participant focused on the muscle groups being used. The exercises performed were jaw clenching, fist clenching, pushing hands down onto thighs, pushing palms together, squeezing thighs together, and pressing the soles of the feet down into the floor. Each exercise was performed for one minute and the remainder of the ten minutes was spent introducing the exercises and encouraging relaxation and abdominal breathing between the exercises. The body scan instructions drew attention to sensations in different areas of the body during inspiration and expiration. Participants were advised that these activities might help reduce urges to smoke and were encouraged to use one or both activities every time they where afflicted by urges to smoke. They were given step-by-step instructions of how to transfer the files to .mp3 players or burn them onto CDs. This would allow participants to do the exercises while listening to the instructions, but it was possible for participants to practice these at home and remember how to do them for use when the need arose. Participants in the control group were given no exercises or activities and the email explained that they were in the control group and were thanked for completing the weekly questionnaires. We did not obtain data on whether control participants used exercises, so it is possible that they did the exercises at home anyway. This is unlikely, however, because isometric exercise and body scan are not commonly used in the UK and are unknown to smoking cessation therapists.

At the first recruitment visit, researchers obtained basic demographic and smoking data. At each of the four subsequent weekly clinic visits, we obtained data from the clinic records on smoking in the past week and exhaled carbon monoxide (CO) reading to validate abstinence (defined as <10 parts per million). Clinic staff requesting these data were blind to allocation. Participants filled out a self-completion questionnaire comprising the Mood and Physical Symptoms Scale (MPSS)[15], which measures urge frequency and intensity and withdrawal symptoms, and participants in the intervention group completed questions on the use of isometric and body scan exercises and the perceived usefulness of these. In keeping with the standard instructions of the MPSS, urge intensity and withdrawal were not measured while smoking, but withdrawal symptom severity was and weekly measures were measured adjusted for baseline measures. On the fourth week, the questionnaire for the intervention group also included an overall assessment of the value of the exercises (Table 1).

**Table 1:** Questions used to assess response to body scan and isometric exercises

Question		Response options
Weekly assessment		
1	If you have experienced urges to smoke, how often have you done the isometric exercises?	Almost always with every urge, a lot of the time, some of the time, a little of the time, never used it
2	If you have used the isometric exercises, how have they affected the urges to smoke?	Reduced the urges, slightly reduced the urges, did not affect the urges, slightly increased the urges, increased the urges
3	If you have experienced the urges to smoke, how often have you done a body scan?	Almost always with every urge, a lot of the time, some of the time, a little of the time, never used it
4	If you have used the body scan, how has doing the body scan affected your urges to smoke?	Reduced the urges, slightly reduced the urges, did not affect the urges, slightly increased the urges, increased the urges
Overall evaluation		
	Will you carry on using the isometric exercises or body scan?	Yes/no
	Did you find the isometric exercises and body scan easy to fit into your life?	Easy to fit in my life almost always, easy to fit in my life most of the time, sometimes easy to fit in my life, rarely fitted into my life, never fitted into my life
	Would you recommend isometric exercises or body scan to a friend trying to stop smoking?	Yes/no

We did not seek a particular number of participants because this was a pilot study, though advice on pilot studies suggests that about 40 participants is a reasonable number[16]. There was no prospect of running a trial large enough to examine the effectiveness of the techniques. We sought instead to examine whether it was possible for quitters in typical cessation clinics to use the techniques and to discover their reactions to the techniques. We used the most practical method of distributing the instruction recordings and the instruction sheet. We compared the proportion achieving 4-week prolonged abstinence measured according to the Russell standard, meaning a 2-week grace period was allowed and dropouts were counted as treatment failures[17]. We calculated the rate ratio (RR) and 95% confidence intervals (CI) for these proportions. We compared individual items measured on the MPSS or other rating scales with Mann-Whitney-U test. Composite scores from the MPSS were compared with a t-test, calculating 95%CI. In keeping with consensus guidelines, we analysed withdrawal phenomena only in those who were abstinent, defined as an exhaled CO<10 ppm[18].

## Results

40 participants were recruited between January and February 2007. Their smoking behaviour was similar to typical smokers in NHS treatment clinics, except that they were somewhat younger on average than typical smokers in cessation clinics (Table 2)[19,20].

**Table 2:** Baseline characteristics of participants by trial arm

	Intervention N = 20	Control N = 20
Age years Mean (SD)	32.5 (12.6)	36.5 (10.9)
Gender N (%) female	10 (50%)	11 (55%)
Cigs/day Mean (SD)	18 (7)	20 (10)
FTND Mean (SD)	4.9 (2.2)	5.4 (2.4)
Baseline CO ppm Mean (SD)	23 (18)	29 (9)

^1 ^Fagerstrom Test for Nicotine Dependence[24], scored 0 (least dependent) to 10 (most dependent)

### Process measures

Emails were sent to all participants in both groups and 17 people (85%) in the intervention group reported receiving it, one did not get it, and two missed the second clinic visit when these data were collected. All 20 participants (100%) in the control group received the email. Ten of the 20 people in the intervention group (59% of those who received the email) printed the isometric exercise visual instructions, and of these 9 (90%) carried the instructions on their person. Thirteen (65%) of the intervention group owned an .mp3 player, and of these 13, one person attended no clinics after baseline and data on downloading the files were unavailable. Six (46%) of the .mp3 owners did not download the body scan and isometric .mp3 files and six did.

Each week, around three quarters of participants used the isometric exercises and around half used body scan (Table 3). This is more than the number that downloaded the mp3 files, implying people were using the interventions without hearing the instructions at the time of using the techniques. On average, when participants had an urge, they used the techniques 'some of the time' and reported that they 'slightly reduced' their urges to smoke in such circumstances. More people tended to use the isometric exercises compared with the body scan.

**Table 3:** Process measures of the use and perceived value of isometric exercises and body scan for coping with urges to smoke

		Isometric			Body scan		
		
	Number present	N (%) using	Frequency of use with urges Q1 Median (interquartile range)^1,3^	Effect on urges Q2 Median (interquartile range)^2,3^	N (%) using	Frequency of use with urges Q3 Median (interquartile range)^1,3^	Effect on urges Q4 Median (interquartile range^2,3^)
Week 1	17	11 (65)	3 (2 to 3)	2 (1 to 2)	7 (41)	3 (3 to 4)	2 (2 to 2)
Week 2	13	10 (77)	3 (2 to 4)	2 (1.75 to 2)	8 (61)	2 (1 to 2.75)	2 (1 to 1.75)
Week 3	11	10 (91)	3 (2.75 to 3.25)	2 (1.75 to 2)	8 (73)	3 (3 to 3)	2 (1 to 2)
Week 4	16	13 (81)	3 (3 to 4)	2 (2 to 2)	8 (50)	3 (3 to 3.75)	2 (1.25 to 2)

1 Scored 1-almost always, 2-a lot, 3-sometimes, 4-a little, 5-never

2 Scored 1-reduced urges, 2-reduced slightly, 3-no effect, 4-slightly increased, 5-increased

3 Excludes those who did not report using method

### Effects on abstinence

Nine of the 20 (45.0%) in the intervention group achieved abstinence at 4 weeks, compared with 11/20 (55.0%) in the control group. The RR (95%CI) was 0.82 (0.44–1.53), a risk difference (95%CI) of -10% (-41% to 21%). These abstinence rates are similar to those achieved by the NHS Stop Smoking Service[21].

### Effects on withdrawal

There were no statistically significant differences in the intensity of withdrawal symptoms or in urges to smoke between intervention and control groups (Table 4).

**Table 4:** Effects of intervention on nicotine withdrawal symptoms and urges to smoke

	Intervention Mean (SD)	Control Mean (SD)	Difference (95%CI) intervention minus control^1^
Withdrawal symptoms			
MPSS-M baseline	8.9 (3.3)	10.9 (4.0)	
MPSS-M week 1	10.1 (3.3)	10.4 (2.5)	-0.6 (-3.4 to 2.9)
MPSS-M week 2	9.4 (3.1)	10.6 (3.6)	-0.1 (-2.6 to 2.4)
MPSS-M week 3	8.5 (2.1)	10.6 (2.9)	-1.8 (-5.3 to 1.8)
MPSS-M week 4	8.6 (2.7)	10.8 (3.4)	-1.1 (-4.5 to 2.3)
Urges to smoke			
MPSS-C week 1	7.1 (1.8)	7.0 (2.2)	0.1 (-1.6 to 1.8)
MPSS-C week 2	7.6 (1.8)	7.2 (2.6)	0.4 (-1.6 to 2.3)
MPSS-C week 3	7.0 (1.4)	6.3 (2.8)	0.7 (-2.0 to 3.5)
MPSS-C week 4	8.3 (2.0)	7.3 (2.5)	1.0 (-1.4 to 3.4)

1 Adjusted for baseline for MPSS-M values but not MPSS-C in accord with instructions for the scale

### Overall evaluation by intervention participants

Of the 16 participants at week 4, 13 (81%) intended to carry on using the isometric exercises and 4 (25%) intended to continue the body scan. Only two (13%) people reported that the exercises did not fit in with their life and the typical response was that it was 'sometimes' easy to incorporate these activities into life. Thirteen (81%) would recommend the isometric exercises to someone stopping smoking and 8 (50%) would recommend the body scan.

## Discussion and conclusion

Among smokers with an email address who received instructions to use isometric exercises and body scan, most of them tried it, most persisted with it, and most would recommend the techniques to others trying to stop smoking. Isometric exercises were more popular than was body scan. Although participants reported that using these techniques were slightly helpful in reducing urges to smoke, there was no evidence that such interventions reduced intensity and frequency of urges to smoke or withdrawal symptoms, as measured by the MPSS. The study was not large enough to detect differences in short-term abstinence and none were found.

This study was designed to test isometric exercises and body scan in a routine smoker's clinic. Our only stipulation was that participants had an email address. Of these, most had an .mp3 player but fewer than half of those with one downloaded the instructions onto it. This probably affected the use of body scan, which may be difficult to do without hearing the instructions to do it until it has become a familiar routine. This may be due to simple inertia. The price of .mp3 players is now so low that they could easily be preloaded with a variety of files and given as part of treatment programmes, and thus cut out the requirement for individuals to download their own files. We adopted a pragmatic design trial[14], using the simplest method we could to distribute the intervention and we did not train the smoking cessation clinic staff to encourage the use of the intervention. We also did not restrict the medication choices made in the clinic, which would introduce an element of random variation making it harder to detect an effect of the exercises on outcomes. That around three quarters of the population used these techniques to control their urges to smoke in such a setting shows the appetite of quitters for simple evidence-based techniques to help control urges to smoke and perhaps explains the persistence of the 4Ds despite lack of evidence that they are effective. Indeed, a very intensive intervention which advised 45 minutes daily of mindfulness meditation and a 7-hour meditation on quit day was highly regarded by many participants and produced a high quit rate[22].

The trial was small and consequently there were some minor imbalances between the arms, generally appearing to favour the intervention over the control arm. This may have exaggerated slightly the point estimate of the effect of the intervention. Even considering this, the confidence intervals round the effect size encompassed more than a 50% increase in rate of abstinence. This is as large as the effect of individual behavioural support[3]. Overall, however, despite the lack of effect on withdrawal and abstinence, we regard this study as encouraging of a larger trial to examine the efficacy of the two behavioural interventions we tested. The interventions were positively regarded and used by most people to cope with at least some of the urges to smoke and they perceived these as slightly helpful. Although NRT can reduce urges to smoke, it does not 'kick in' until about 10–15 minutes after the urges strike[23], whereas isometric exercises and body scan can be done immediately. It might therefore make sense to develop an intervention that involves using an acute form of NRT on feeling the urges and then commence one of these exercises. A further trial incorporating these kinds of behavioural interventions is needed.

## Competing interests

The authors declare that they have no competing interests.

## Authors' contributions

All authors contributed to the design of the study. JB provided clinical supervision. LA, NP, LS, and SS recruited the participants and collected the data. JB provided the clinical outcome data. PA analysed the data and drafted the initial manuscript and all authors contributed to improving it.

## Pre-publication history

The pre-publication history for this paper can be accessed here:



## References

[B1] Aveyard P, West R (2007). Managing smoking cessation. BMJ.

[B2] Hajek P (1989). Withdrawal-oriented therapy for smokers. Br J Addict.

[B3] Lancaster T, Stead LF (2005). Individual behavioural counselling for smoking cessation. Cochrane Database Syst Rev.

[B4] Stead LF, Lancaster T, Perera R (2003). Telephone counselling for smoking cessation. The Cochrane Database of Systematic Reviews: Reviews 2003.

[B5] Stead LF, Lancaster T (2005). Group behaviour therapy programmes for smoking cessation. Cochrane Database Syst Rev.

[B6] Hughes JR, Stead LF, Lancaster T (2007). Antidepressants for smoking cessation. Cochrane Database of Systematic Reviews: Reviews 2007.

[B7] Stead LF, Perera R, Bullen C, Mant D, Lancaster T (2008). Nicotine replacement therapy for smoking cessation. Cochrane Database of Systematic Reviews: Reviews 2008.

[B8] Killen JD, Fortmann SP (1997). Craving is associated with smoking relapse: findings from three prospective studies. Experimental and Clinical Pschopharmacology.

[B9] Bittoun R (2003). The four Ds deconstructed. Aust N Z J Public Health.

[B10] Taylor AH, Ussher MH, Faulkner G (2007). The acute effects of exercise on cigarette cravings, withdrawal symptoms, affect and smoking behaviour: a systematic review. Addiction.

[B11] Ussher M, West R, Doshi R, Kaur Sampuran A (2006). Acute effect of isometric exercise on desire to smoke and tobacco withdrawal symptoms. Human Psychopharmacology: Clinical and Experimental.

[B12] Cropley M, Ussher M, Charitou E (2007). Acute effects of a guided relaxation routine (body scan) on tobacco withdrawal symptoms and cravings in abstinent smokers. Addiction.

[B13] MRC (2000). A framework for development and evaluation of RCTs for complex interventions to improve health. http://www.mrc.ac.uk/Utilities/Documentrecord/index.htm?d=MRC003372.

[B14] Roland M, Torgerson DJ (1998). Understanding controlled trials. What are pragmatic trials?. BMJ.

[B15] West R, Hajek P (2004). Evaluation of the mood and physical symptoms scale (MPSS) to assess cigarette withdrawal. Psychopharmacology (Berl).

[B16] Lancaster GA, Dodd S, Williamson PR (2004). Design and analysis of pilot studies: recommendations for good practice. Journal of Evaluation in Clinical Practice.

[B17] West R, Hajek P, Stead L, Stapleton J (2005). Outcome criteria in smoking cessation trials: proposal for a common standard. Addiction.

[B18] Shiffman S, West RJ, Gilbert DG (2004). Recommendation for the assessment of tobacco craving and withdrawal in smoking cessation trials. Nicotine & Tobacco Research.

[B19] Aveyard P, Brown K, Saunders C, Alexander A, Johnstone E, Munafo M (2007). A randomised controlled trial of weekly versus basic smoking cessation support in primary care. Thorax.

[B20] Aveyard P, Johnson C, Fillingham S, Parsons A, Murphy M (2008). A pragmatic randomised controlled trial of nortriptyline plus nicotine replacement versus placebo plus nicotine replacement for smoking cessation. BMJ.

[B21] Judge K, Bauld L, Chesterman J, Ferguson J (2005). The English smoking treatment services: short-term outcomes. Addiction.

[B22] Davis J, Fleming M, Bonus K, Baker T (2007). A pilot study on mindfulness based stress reduction for smokers. BMC Complementary and Alternative Medicine.

[B23] Shiffman S, Shadel WG, Niaura R, Khayrallah MA, Jorenby DE, Ryan CF (2003). Efficacy of acute administration of nicotine gum in relief of cue-provoked cigarette craving. Psychopharmacology (Berl).

[B24] Heatherton TF, Kozlowski LT, Frecker RC, Fagerstrom KO (1991). The Fagerstrom Test for Nicotine Dependence: a revision of the Fagerstrom Tolerance Questionnaire. British Journal of Addiction.

